# Towards school-based mental health programs in Nigeria: the immediate impact of a depression-literacy program among school-going adolescents and their teachers

**DOI:** 10.1186/s13034-022-00503-9

**Published:** 2022-08-23

**Authors:** Olayinka Atilola, Olatunde Ayinde, Felix-Kingsley Obialo, Sunday Oladotun Adeyemo, Dapo Adegbaju, Robert Anthony

**Affiliations:** 1 Department of Behavioral Medicine, Lagos State University College of Medicine Ikeja, Lagos, Nigeria; 2 Department of Psychiatry, College of Medicine, University of Ibadan, Ibadan, Nigeria; 3 Center for Creativity and Entrepreneurial Studies, Dominican University Ibadan, Ibadan, Nigeria; 4 Department of Psychology, Olabisi Onabanjo University Ago-Iwoye, Ago-Iwoye, Nigeria; 5 Federal Neuro-Psychiatric Hospital Yaba, Lagos, Nigeria; 6 Adolescent Wellness, Inc, Naples, FL USA

**Keywords:** Depression, Depression literacy, Adolescents, School mental-health

## Abstract

**Background:**

Depression-literacy, which is the foundational requirement for symptom recognition, positive attitude and help-seeking, is poor among adolescents in Nigeria. This study, therefore, aims to determine the impact of a school-based training program on depression-literacy among a cohort of high-school students and their teachers in South-West Nigeria.

**Methods:**

An adapted version of the Break Free from Depression, a 4-module depression awareness curriculum for staff and students, was implemented among students and their teachers. Paired-sample T-test was used to assess the domain-specific (knowledge, attitude, and confidence) impact of the training by comparing the baseline and immediate (within the week of the training) post-scores.

**Results:**

A total of 3098 students and 294 teachers from 21 schools across three states in South-West Nigeria successfully completed the training. There was a significant positive difference (p < 0.05), at post-test, in the knowledge, attitude, and confidence among the students. The same was observed among teachers except for attitude where positive change did not reach significant level (p = 0.06). When statistically significant, the calculated effect size (eta squared) was highest for knowledge (students: 0.07, p = 0.001; teachers: 0.08, p < 0.000) and least for attitude (students: 0.003, p = 0.002 teachers: 0.085, p = 0.06). Multiple regression analyses result showed that the level of pre-scores predicted the magnitude of change in all domains of depression-literacy (p < 0.05) after controlling for age, gender, and type of school among the students, but not for teachers.

**Conclusions:**

School-based depression-literacy programs can lead to significant positive change in knowledge, attitude, and confidence of students and teachers.

## Background

Data from the Global Burden of Disease (GBD) studies have shown that mental disorders contributes significantly to disability among children and adolescents and, in fact, these disorders are likely to rank high among major child and adolescent public health challenges of the twenty-first century [1]. Among the common mental disorders that afflict children and adolescents, GBD data also establish depression as ranking highest in terms of burden [2]. Though prevalence rates vary from one region of the world to another, one cross-national study which sampled adolescents from more than ten countries across the world reported that one out of ten adolescents have clinically significant symptoms of depression [3]. In Nigeria, despite varying prevalence rates, several studies conducted in different parts of the country, have established that depression is common among children and adolescents. For instance, in South-West Nigeria, a study conducted among school-going adolescents in Ibadan identified mild to moderate depression in nearly a quarter (23.8%) and severe depression in 5.7% of respondents [4]. Oderinde et al. also reported a prevalence rate of 16.3% among a sample of school-going adolescents in Ido-Ekiti [5]. Adewuya & Ologun reported a lower but equally high prevalence rate of 9.0% in a different sample of adolescents sampled from schools in Ile-Ife [6]. Studies conducted in other regions of the country also established a significantly high burden of adolescent depression [7, 8].

When left untreated, depression among children and adolescents is associated with significant short/medium-term risks such as missed school days and suicidal behaviours [9] and long-term negative consequences such as persistent recurrence, poorer physical health morbidity, and chronic alcohol abuse [10–12]. It is therefore appropriate that investment in strategies to reduce the burden of depression at key developmental phases across lifespan, particularly during adolescence, is being seen as a global public health goal [13]. Fortunately, there is robust evidence for safe, effective, and affordable pharmacologic and non-pharmacologic interventions for adolescent depression worldwide (14–16). However, for a variety of reasons, there has been significant barriers to mental health services, including treatment for depression, among adolescents worldwide, worse in low- and middle-income countries, and as such, creating a treatment-gap [17].

Interventions to reduce adolescent depression treatment-gap must acknowledge the fact that access to treatment of depression among adolescents is, among other factors, a function of the degree of recognition of the constellation of experiences which characterizes the syndrome of depression, as a health concern which can be understood, prevented, or treated [18]. In other words, adolescent depression-literacy must improve as a pre-condition for the success, in terms of uptake, of available intervention strategies. This is because poor depression-literacy, whereby depressive symptoms were interpreted as normal inconveniences of puberty or a personality trait, has been identified as one of the potential barriers to help-seeking among depressed adolescents [19]. Furthermore, there is preliminary evidence, from around the world, that school-based mental health awareness programs which targets the provisioning of accurate information about the nature of and services for mental health conditions to parents, teachers, and students, can and does lead to improved symptom recognition and uptake of mental-health services among school children [20–22]. Aside from being the foundational component upon which additional structures for promoting mental-health, such as symptom recognition, tolerant attitude, and improved service utilization, can be built [23]; improved mental-health literacy of students also come with additional benefits such as reduced prejudice and stigma, more positive emotional response towards peers who may be experiencing depression [24], and positive personal mental health in general [25, 26].

Recent research in Nigeria has shown that the health-education curriculum of Nigerian elementary and secondary schools focused on hygiene and environmental health with hardly any meaningful mental-health content [27]. Hence, knowledge of school-going adolescents, and indeed their teachers, about mental illness in general, is limited [28, 29]. By extension, attitude of school-going adolescents towards individuals with mental disorders has been shown to be negative in one study and reflected desire for social distance [30]. There have been very few school-based short-term training programs aimed at improving mental-health literacy of students in Nigeria in recent times. Such efforts have yielded immediate and sustained positive change in aspects of general mental-health literacy among small samples of children and adolescents attending elementary and secondary schools in the country [31, 32]. However, there has not been any documented intervention (school- or community-based) aimed at improving depression-literacy in particular among adolescents in Nigeria, and indeed in sub-Saharan Africa. This is despite the fact that more recent research, which examined depression-literacy specifically among school-going adolescents, found that only a paltry 4.8% of the sample correctly identified a depression vignette while another paltry 1.5% recommended professional help as a treatment option [33]. Furthermore, the few earlier intervention studies for general mental-health literacy conducted in Nigeria did not involve teachers. Teachers are equally important members of the school environment and mental-health literacy has been shown to be equally poor among them [28, 29]. Improving the mental-health literacy of teachers alongside the students has been described as “critical” to the success of school-based mental-health promotion and intervention [34].

From the foregoing, it is obvious that more mental-health literacy programs are needed in Nigeria, and indeed Africa, especially large-scale intervention programs focussing on a specific mental-health problem such as depression. The present study, therefore, aimed to conduct an implementation of a standardized depression-literacy program among a large and representative sample of school-going adolescents and their teachers in Nigeria. In this paper, we report the immediate post-intervention impact, in terms of satisfaction with the program, the knowledge gained and the socio-demographic factors that independently influenced it.

## Methods

### Design and setting

The quasi-experimental post–pre interventional study was conducted in secondary schools within the three states of Oyo, Ogun, and Osun in South-West Nigeria. The three states were picked based on convenience, though their combined population represents about 10% of the Nigerian population.

### Sampling

The data analysed for this study came from an ongoing community project, carried out in conjunction with the Rotary International, on which the authors served as technical and scientific partners. By the original design of the project, a convenient sample of schools from the three contiguous southwestern states of Oyo Ogun, and Osun in Nigeria were selected, to reflect adequate representation of rural and urban, as well as private and public schools. In each selected school, school counsellors contacted all the students in each class to participate in the project. An explanatory note describing the project was sent to their parents. All contacted students whose parents provided written informed consent and who assented to participate were then included in the project. All the teachers in each of the selected schools were contacted to participate in the project through an explanatory note with an attached consent form. All teachers who returned signed consent forms were recruited into the project.

### Procedures

#### The intervention

The intervention was adapted from the Break Free from Depression, a 4-module Depression Awareness Curriculum for Staff and Students developed by the Boston Children Hospital Neighborhood Partnerships [35]. The manual was developed from an initial depression tool-kit for schools [36], and later further enhanced through several years of working with school children with depression and getting feeds-back from them. The present version has been used in several schools in the United States of America (USA) and in a few middle-income countries such as India. It is currently included in the best-practice registry of the Suicide Prevention Resource Center in the USA. The manual has three main components: The Staff (teachers and other administrative/ support staff) Training, Parent Events, and the implementation of the Student Curriculum. The teacher and parent components, with slight differences in the content and language to suit the audience, are both essentially geared towards educating the teachers/parents about the rationale for a depression awareness program in schools, nature and dynamics of depression and suicidal behaviors among adolescents, and ways to help and/or refer students/wards experiencing these symptoms. The student curriculum, on the other hand, has four modules originally. The first module (Module 1) is essentially an overview of the facts about the nature, sign, and symptoms of depression and suicidality, including efforts to dispel local myths about the condition. The second module (Module 2) is a film documentary aimed at ‘bringing alive’, in audio-visual format, the symptoms, stigma, risk factors, and coping strategies for depression. The third module (Module 3) is essentially a small-group debriefing session in which participants are encouraged to apply the knowledge gained from Module 1 to a set of pre-written case studies of depression and suicidality among hypothetical teenagers. The module also covers the key warning signs of suicide, basic creative problem-solving skills, and additional resources that can be accessed if further information is needed. The fourth module (Module 4) is an open discussion on self-help strategies for depression and the steps to be taken to access or help a friend to access additional mental-health services. It involves the use of presentations and role plays.

#### The pilot implementation

##### Preparation

The entire school community, including students, teachers, parents’ associations, and school authorities were briefed about the program and the potential benefits. A lead-staff, often the school counsellor, was identified to be the internal coordinator for the project in each school. The internal coordinator liaised between the program team and the school community, helped to provide answers to preliminary inquiries, addressed logistical issues in the course of the program, and served as the continuous link between the program team and the school during and after the program.

##### Adaptation

The original version of the depression awareness curriculum was adapted to suit the culture, resource-environment, and the available funds for the project. Firstly, the entire curriculum for the staff training, parents’ event, and student curriculum was adapted to be more relevant to the culture of south-west Nigeria. This was in consideration of the observations from systematic review of qualitative studies around the world, which have established cultural differences and nuances in the perception of the nature of and explanatory model for mental disorders, including depression [37]. The adaptation was done by a panel, comprised of the investigators, who are mental-health practitioners with wide experience with the local realities of depression among adolescents in the region, particularly with reference to peculiarities in risk factors, coping strategies, idioms of expression of psychopathology, and nuances in explanatory models of depression. The panel reviewed the entire curriculum and the case studies for appropriateness and cultural relevance. Firstly, at the start of implementation of Module 1, we gave an overview of what ‘depression’ was and enquired what local language or idiom is used to expressed such state. This was done with the intention to use an appropriate idiom of expression for depression in the locality and to differentiate it from other mental illnesses such as *vagrant psychosis,* which often typify mental illnesses among community dwellers in the study setting. Other minor changes were made to the content of the curriculum and case studies based on a consensus of the experts. For instance, though the program implementation was done in English language, the local language was interspersed from time to time to explain unfamiliar vocabularies in the Modules. Words such as “hopelessness”, “coping strategies”, “emotional pain” and “panic attacks” were broken down into simpler words or local idioms were used to replace them. The word “black” which was used to describe the experience of being a minority in one of the case studies was replaced with another prejudiced ethnic minority group in Nigeria, which the adolescents are familiar with. In addition, all the foreign names of hypothetical case-study individuals in Module 3 were changed to local names to enhance familiarity. We were not able to implement Module 2, that is, the film documentary part for the students. This was due to two main reasons. The first reason was the impracticability of culturally adapting the contents of the film documentary. The second reason was the logistic difficulties of airing the documentaries in some rural locations. Rather, we made sure to enrich the contents of Modules 1 and 3 to adequately cover the description of the symptoms and signs of depression with more illustrative examples.

##### Implementation proper

All trainings were led by a member of the team supported by all other members of team. The training-lead is a doctoral candidate in clinical psychology (SOA) with experience in conducting psycho-educational programs in schools. He is also fluent in both English and Yoruba, the local language of the region. All trainings were conducted in simple English, with occasional use of Yoruba, the local language to emphasize some points or concepts that were difficult to capture in English. The staff training was the first event, and it took place in batches in one of the school classrooms in each participating school. It involved teachers only. All available and consenting teachers took part. The parents’ event was a single-day event. The parents were recruited through the schools’ Parent Teacher Association. This was also a single-day event and all recruited parents who showed-up were asked to participate after giving their consent. The Module 1 and 4 of the student curriculum training was done in a single group of no more than 50 participants in one of the spacious school hall/classrooms. The Module 3 was implemented in small sub-groups of about 10 participants.

##### Evaluation

Pre- and post-survey was administered to both student- and teacher-participants as part of the training. The pre-survey was a 5–10 munite, 22-item survey which is part of the original Break Free from Depression Modules. The surveys measures respondents’ knowledge (8 items) and attitude (5 items) about depression, as well as their level of confidence (9 items) in responding to a situation related to depression on a 4-point Likert scale ranging from strongly disagree to strongly agree. The pre-survey was administered before the start of the program. The post-survey, which was administered within the week of completing the training, was a replica of the pre-survey but, in addition, had extra sections assessing, on similar 4-point Likert scale, the overall satisfaction of respondents with the training. A single ‘Yes’ or ‘No’ question about whether respondent had referred someone, including themselves, to an adult or professional (as the case may be for students and teachers respectively), as a result of the training they had received from the program, was also added to the post-survey. All pre-and post-surveys for each respondent were matched and linked with a code. In the final analysis, respondents who strongly agreed/ agreed were grouped together as one group while those who disagreed/ strongly disagreed were also grouped together. Items that were negative on the attitude scale were reversed scored in such a way that higher scores indicated better attitude.

### Ethical consideration

We followed the ethical guideline of ensuring voluntariness, confidentiality, and non-malfeasance in the conduct of the research. The purpose of the research and any risk involved was explained to all would-be participants. Appropriate permissions were obtained from the local education authorities and the heads of each school. Written informed consent was obtained from parent and teachers, both for their own participation and that of their ward/students. The entire protocol of the project was also submitted to and approved by the local ethical review board. As additional benefit of participating in the program, a referral protocol for students who either sought help from the research team or were referred by friends or teachers was developed and implemented in a safe and confidential manner throughout the period of the project.

### Statistical analyses

Data was managed and analyzed using the Statistical Package for Social Sciences (SPSS) version 20.0. Descriptive statistics was used to present the age, gender, and other socio-demographic characteristics of participants across study sites. Paired-sample T-test was used to assess the overall and domain-specific (knowledge, attitude, and confidence) impact of the training within the same week of intervention by comparing the mean scores for the pre- and post-tests. A measure of effect sizes was obtained by dividing the overall mean difference in pre- and post-scores with the standard deviation. Multiple regression analysis, one model for the students and one model for the teachers, was used to determine the independent socio-demographic facilitators or barriers to improved literacy domains among the respondents. In the models, the dependent variable was the post intervention scores on the different domains as continuous variables. The independent variables were the socio-demographic data such as age, gender, type of school (public vs. private) and the baseline pre-test scores of the domain in question. The baseline pre-test score was included to correct for initial differences in the baseline scores of respondents. The model summary and the R-square for the model was presented after checking to make sure that standard assumptions such as linearity, collinearity, and homoscedasticity were not violated. Finally, the responses to the section on satisfaction with the program on the post-test was analyzed. The 4-point Likert responses was collapsed into a ‘yes’ (strongly agree and sort-of agree) and ‘no’ (strongly disagree and sort-of disagree) dichotomy. The proportion of respondents who reported satisfaction (yes response) for each of the satisfaction questions on the post-test were also recorded.

## Results

The Break Free from Depression training modules, as modified, was successfully implemented in 21 public and private secondary schools across South-West Nigeria. The school authorities, teachers and students were mostly cooperative and supportive. The post-intervention results for the students and the teachers are presented separately.

### Students

A total 3,098 secondary school students sampled from 21 public and private secondary schools in South-West Nigeria completed the training program. The mean age was 16.4 (± 1.58; range 11–19) years. There were more females (n = 2049; 66.1%) than males. A slight majority of the students were from public secondary schools (n = 1769, 57.1%). As shown in Table 1, there was a significant positive difference (p < 0.05), at post-test, for each of the three domains of training, that is: knowledge of, attitude towards, and confidence in handling depression and suicidality among the students. The magnitude of the effect size (eta squared) was highest for knowledge (0.07, medium effect) and least for attitude (0.003, negligible). Multiple regression analyses result showed that the only statistically significant predictor of post intervention knowledge score was the level of pre-intervention knowledge scores among the students (see Table 2). Aside the pre intervention attitude scores which predicted post-intervention attitude score (β = 0.256, p < 0.001), students of private institutions had statistically significant better scores on attitude (β = 0.170, p < 0.001) compared with those in public schools. Age, sex, type of school, and pre-intervention level of self-reported confidence all predicted post-intervention confidence score in handling issues related to depression and suicidality. In the model, older respondents (β = 0.083, p < 0.001), females (0.460, p < 0.001), private-school students (β = 0.287, p = 0.002), and those with higher pre-intervention confidence scores (β = 0.286, p < 0.001) all had significantly higher post-intervention confidence scores. Furthermore, at the completion of the program, almost three-quarters (71%, n = 2195) of the students who participated in the training agreed that the program was a good way to learn about depression and suicidality and would, therefore, recommend the program to a friend. Half of the participating students (50.9%, n = 1578) admitted to knowing a peer (including themselves) who sought help or advice about depression or suicidality from an adult, based on the lessons learnt from the training program. An overwhelming majority (82.5%, n-2552) expressed willingness to be a peer-trainer on the program if such opportunity was available in the future.

**Table 1:** Paired Sample T-test for the post and Pre-test comparison for both students and teachers

	Paired differences								
	Mean difference	Std. deviation	η^2^ (Eta square)	Std. error mean	95% CI of the difference lower upper		t	df	Sig. (2-tailed)
Students (*n* = 3098)									
Post-knowledge	0.458	1.663	0.070	0.030	0.339	0.517	15.327	3097	**0.001**
Post-attitude	0.081	1.449	0.003	0.026	0.030	0.132	3.113	3097	**0.002**
Post-confidence	0.514	2.822	0.030	0.051	0.414	0.613	10.129	3097	**0.001**
Teachers (*n* = 234)									
Post-knowledge	0.521	1.825	0.081	0.119	0.286	0.756	4.369	233	** < 0.001**
Post-attitude	0.534	4.237	0.085	0.277	−0.011	1.079	1.928	233	0.06
Post-confidence	2.290	8.086	0.802	0.528	1.249	3.332	4.333	233	** < 0.001**

*df* degree of freedom, *t* student T-test

**Table 2:** Predictors of students’ post-intervention knowledge of, attitude towards, and confidence about handling depression

Independent variables	β	t	sig	Confidence interval	
				Lower bound	Upper bound
Model 1 (dependent variable: post-knowledge)					
Age	−0.017	−0.987	0.324	−0.042	0.014
Sex (male coded 1, female 0)	−0.002	−0.111	0.912	−0.099	0.089
School type (public coded 1 vs. private coded 0)	−0.059	−1.289	0.198	−0.149	0.031
Knowledge (pre-scores)	0.183	10.342	**<0.001**	0.140	0.206
Constant	2.896	19.884	0.000	4.607	5.614
Model 2 (dependent variable: post-attitude)					
Age	0.002	−0.168	0.866	−0.027	−0.023
Sex (male coded 1, female coded 1)	0.010	0.231	0.818	−0.074	0.094
School type (public coded 1 vs. private coded 0)	−0.256	−6.230	**<0.001**	−0.336	−0.175
Attitude (pre-scores)	0.170	9.437	**<0.001**	0.336	−0.175
Constant	2.383	10.85	0.000	1.952	2.183
Model 3 (dependent variable: post-confidence)					
Age	0.083	4.859	**<0.001**	0.075	0.177
Sex (male coded 1)	−0.460	−5.204	**<0.001**	−0.653	−0.277
School type (public coded 1 vs. private coded 0)	−0.287	−3.173	**0.002**	−0.433	−0.102
Confidence (pre-scores)	0.286	16.695	**<0.001**	0.260	0.330
Constant	3.092	6.966	0.000	2.221	3.962

Model 1; R^2^ = .035, F = 27.98, **p < 0.001**

Model 2; R^2^ = .042, F = 34.55, **p < 0.001**

Model 3; R^2^ = .10, F = 87.00, **p < 0.001**

*β* standardized co-efficient; *t *student T-test

### Teachers

A total 234 teachers from the 21 participating schools completed the training program. The mean age of the teachers was 39.8 years (SD: 9.97 years, range: 23 to 68 years). There were more females (n = 134;57.3%) than males. Majority of the teachers were from private secondary schools (n = 146; 62.4%). As shown in Table 1, self-reported knowledge of and confidence in handling issues related to depression and suicidality improved significantly (p < 0.05) and at roughly the same level of magnitude (η^2^ = 0.081 and 0.080 respectively), which is of medium effect size, among the teachers at post-test. Though there was a demonstrable positive change in mean attitude scores post-intervention, the difference did not reach statistical significance (p = 0.06). Multiple regression analysis showed that pre-intervention attitude score was the sole predictor (p < 0.001) of the slight positive change in attitude (Table 3). Same regression models for knowledge and confidence did not show any statistically significant predictors of either outcome (Table 3).

**Table 3:** Predictors of teachers’ post-intervention knowledge of, attitude towards, and confidence about handling depression

Independent variables	β	t	sig	Confidence interval	
				Lower bound	Upper bound
Model 1 (dependent variable: post-knowledge)					
Age	0.105	1.562	0.120	−0.004	0.033
Sex (male coded 1, female 0)	0.105	1.584	0.114	−0.073	0.672
School type (public coded 1 vs. private coded 0)	0.001	0.002	0.998	−0.362	0.363
Knowledge (pre-scores)	0.100	0.1434	1.434	0.153	0.037
Constant	4.477	7.142	0.000	3.242	5.712
Model 2 (dependent variable: post-attitude)					
Age	−0.032	−0.491	0.624	−0.018	0.011
Sex (male coded 1, female coded 0)	0.017	0.260	0.795	−0.261	0.341
School type (public coded 1 vs. private coded 0)	0.234	1.573	0.117	−0.059	0.527
Attitude (pre-scores)	0.220	3.490	**0.001**	0.096	0.345
Constant	2.174	5.031	0.000	1.323	3.025
Model 3 (dependent variable: post-confidence)					
Age	0.091	1.382	0.168	−0.099	0.052
Sex (male coded 1)	0.478	1.498	0.135	−0.151	1.107
School type (public coded 1 vs. private coded 0)	0.111	0.036	0.971	−0.599	0.621
Confidence (pre-scores)	0.131	1.961	0.051	−0.001	0.263
Constant	5.187	5.569	0.000	3.352	7.022

Model 1: *R*^2^  0.050, *F* 4.05, *p* **0.003**

Model 2: *R*^2^ = 0.20, *F* = 2.20, *p* = 0.069

Model 3: *R*^2^ = 0.34, *F* = 3.17, *p* = 0.085

*β* standardized co-efficient, *t* student T-test

## Discussion

The Break Free from Depression training module was successfully implemented among a large number of students and teachers spread over large areas in South-West Nigeria. There appears to be more female participation than males. Though data for the school enrolment rate in most part of southern part of Nigeria have shown a higher female enrolment than males [38], the difference is not enough to explain the female preponderance in the present project. It is possible that aside higher female enrolment rate, girls may be more easily recruitable into this kind of project than boys. It is also possible that the recruiters may have been inadvertently biased towards girls as part of the pervading sense of the deliberate need for inclusion of the girl-child in all health programs. Expectedly, there were more student participants from public schools than private schools because public schools are more likely to have a higher student density than private ones. However, there are more teacher-participants from private schools who agreed to participate in the program. This is also understandable as, despite a lower student density compared with public schools, an average private school in Nigeria has higher teacher: student ratio, with teachers that are less burdened with work and with have better incentives. All of these factors  may have enhanced their willingness to participate in an extra-curricular project like the present one.

Turning to the main objective of the project, the successful execution of the modified version of the Break Free from Depression training module in Nigeria is, however, a signal that such large-scale school-based mental-health programs is feasible and potentially impactful. It also confirms earlier needs-assessments in Nigerian schools which have shown that teachers and school administrators recognized the need for and will welcome school-based training programmes that would improve mental-health literacy within the school ecosystem [39, 40]. The overall finding is that the intervention resulted in significant improvement in the three target domains of depression literacy (knowledge, attitude, and confidence) among the students and their teachers. The highest effect size, which is of moderate size, was seen in the knowledge domain, followed by the confidence domain, among the two groups. Other small-scale controlled studies conducted in Nigeria and other parts of Africa have demonstrated improvement in aspects of mental-health literacy after a short training program among students and teachers alike [31, 32, 41].

However, for both groups, that is; students and teachers in the present study, attitude was the least impacted, in terms of both the significance of change and the magnitude of impact. In the present study, the change in attitude for teachers fell short of statistical significance by a few points. While one is inclined to conclude that our intervention improved attitude in students but not teachers, one must be mindful of the difference in sample size of the students compared with teachers in the present project and how this might have affected the statistical power of the analysis and the resulting determination of significance in both groups. The number of students that we were able to include in the study was at least 13 times larger than the number of teachers. It is possible that though the effect size of the change for attitude was the smallest, the sample size of the teachers may have just been below what would have been required to obtain a statistically significant result. The implication is that it is plausible that the intervention was successful in changing knowledge, confidence and attitude for both teachers and students. Suffice to say, however, that there have been mixed results on the impact of mental-health training programs on attitude among students and teachers. Some studies, including ones conducted in Africa, have found significantly improved attitude after mental-health literacy training programs [41, 42]. Other training programmes, however, have observed sustained poor attitude despite intervention [31, 32].

Recent systematic reviews showed that intervention program designs which involved extended contact-time spread over days or weeks and with multiple components (such as survivors lived-experiences and film/ documentaries) had better impact on attitudinal change compared with ultra-short and abridged training programs [43]. Future depression-literacy programs must be mindful of this design consideration. However, such designs must be mindful of the possibility that, in a resource-constrained setting such as the setting of the present project, a lengthier and more involved intervention may also not be as desirable given that it might not be as scalable as the present intervention. Furthermore, compared to the teachers’ group, the students’ group had an almost consistent significantly higher post intervention scores in more domains. This suggests that adolescence may be the golden period for achieving the best results when delivering mental health literacy interventions [44]. Adolescents are probably more likely than adults to find new knowledge interesting and be open minded enough for significant attitudinal change.

In terms of predictors, pre-intervention scores for the three domains of knowledge, attitude, and confidence were predictors of positive change in knowledge, attitude, and confidence, at least among the students. This finding is intuitive because it is expected that those with a pre-existing high-level of knowledge, attitude and confidence may also have better innate abilities to learn or are more likely to connect better with the training. The finding also suggests that mental health literacy interventions should be a continuing exercise, as those who are more likely to benefit from new information are those with better pre-existing information. Additionally, being a student in private school predicted improvements in attitude and confidence, while older adolescents and girls had better improvements on their post-intervention confidence in handling depression and suicidality issues compared with peers, all other factors kept constant. The sociodemographic nuances of impact of interventions are yet to be examined in the adolescent mental-health literacy literature and as such, we are unable to situate these observations. However, suffice to note that students of public and private schools in Nigeria often have wide differences in socio-economic background. Therefore, there might be certain socio-cognitive factors at play which are in favour of the demographics of the private-school students, but which were not measured in the present study. Likewise, there might be certain drivers of age and gender differences in the impact of the training program on attitude and confidence, which were not captured in our design.

Among teachers, improvement in knowledge and confidence was not dependent on age, gender, type of school where they taught, nor their pre-intervention assessment scores. Only their pre-intervention scores on attitude marginally predicted improvement in attitude. Aside from documenting the impact of the program on knowledge, attitude, and confidence, most of the mental-health literacy trainings that have been conducted hardly document the socio-demographic and other predictors of individual benefits. Therefore, there is hardly any previous study to compare these observations with. However, if these findings are replicated in future studies, it may have implications for design of similar interventions for maximal impact. These are areas for future exploration and replication.

There are a few other markers of success of the training program. Up to half of adolescents who participated in the training admitted to knowing someone (including themselves) who has sought help or advise from an adult for depression-related issues within the week of the training. While we did not determine the nature of the inquiry or the quality of the advice received, the finding indicates that the training stimulated conversation around depression and suicidality within the community. Such conversation may eventually lead to help-seeking. Furthermore, close to two-thirds of students who participated in the training program agreed that the program was a good way to learn about depression and suicidality. In the context of general mental-health literacy, school-going adolescents in Nigeria have found other school-based training programs acceptable [32]. This is another indicator of the acceptability of school-based mental-health literacy programs in Nigeria. Lastly, majority of the participating adolescents expressed willingness to be peer-trainers on the program if such opportunities were available. This observation has two different implications. One is that the finding seems to indicate a change in attitude that may not be adequately captured by the items on the survey which are explicitly designed to measure the construct of attitude change. Second is that the suggestion that respondents in this project may embrace peer-led mental-health literacy interventions. Though not yet implemented in the region of Africa, peer-led depression literacy programs has been shown to be acceptable and effective elsewhere [45]. Peer-led programs are viable alternatives to specialist-driven mental-health literacy programs in LMICs, such as Nigeria, where child mental-health specialist practitioners are in short supply and services are constrained [46]. For this reason, peer-led programs are more likely to be scalable in Nigeria than specialist-driven programs such as the present one and should be a future direction. The self-reported enthusiasm of the participating adolescents in the present study to serve as peer-educators is, therefore, encouraging.

The involvement of teachers (and to some extent, parents) is one of the major strengths of the present study. This is because the involvement of teachers and parents is a key addition to primary mental-health interventions, including psychoeducation programs for the adolescents. Though we had to abridge the “Break Free from Depression” depression-literacy program before implementation due to budgetary constraints, this was concurrently done with a cultural adaptation to be more culturally accessible, increase feasibility, and enhance ease of administration. The ability to modify the intervention to improve the fit to the community as well as to fit the resources and funding which are available is of paramount importance in doing effectiveness research. This is especially important to keep in mind with the idea of the intervention being scalable in order to benefit the maximum possible number of youths. Keeping these goals in mind, the fact that the intervention was found to be successful in improving knowledge, confidence and attitudes while being tailored to the needs of the treatment population increases the soundness of the study and its conclusions.

There are, however, a few design limitations of the present study that must be acknowledged. The first is that the schools which served as setting for the study were selected based on convenience. Therefore, the findings may not be easily generalizable. However, the schools were not selected based on any other bias that may have significantly influenced the results. Secondly, due to resource constraints, we were unable to expand the current project to include examination of other potential contributing barriers to service utilization such as stigma of mental-health. However, although we did not measure or intervene in stigma specifically, it is a fact that any effort geared towards improving mental-health literacy, as carried out in this project, will and have been shown to have some spill-over effect on reducing stigma [24]. For instance, an improved attitude, which we demonstrated in this study as one of the outcomes of our intervention, is a proxy for stigma reduction. Future studies may want to include a direct measure of and interventions specific for stigma in this kind of project. For the same reason, we report only the post-intervention outcomes within the week of intervention; we were unable to gather delayed outcome data. As such, we were not able to determine sustained impact. Other small-scale controlled trials of school-based mental-health literacy programs in Nigeria have documented sustained impact up to three months [31, 32]. Despite these limitations, the present study is still the largest school-based depression-literacy training program in Africa to date. Other future directions in subsequent studies will include child-parent co-training or interaction in discussion on depression-literacy program rather than the single-day parent briefing event as we did in the present project. This will enhance a continuous parent–child exchange long after the end of the project. Also, qualitative inquiry can be conducted among the students or teachers will be helpful, especially for the small but significant number who did not express helpfulness of the program as a way of learning about depression and suicidality. Such qualitative engagement will shed more light on better ways of organizing the project for improved acceptability.

## Data Availability

The data and materials that support the findings of this study are available from the corresponding author upon reasonable request.

## Abbreviation

SD: Standard deviation
